# Supplementary figures and images for: Prediction of improvement after extended thymectomy in non-thymomatous myasthenia gravis patients
Source: PLoS One. 2020 Oct 5;15(10):e0239756. doi: 10.1371/journal.pone.0239756 (PMC7535042; doi:10.1371/journal.pone.0239756)

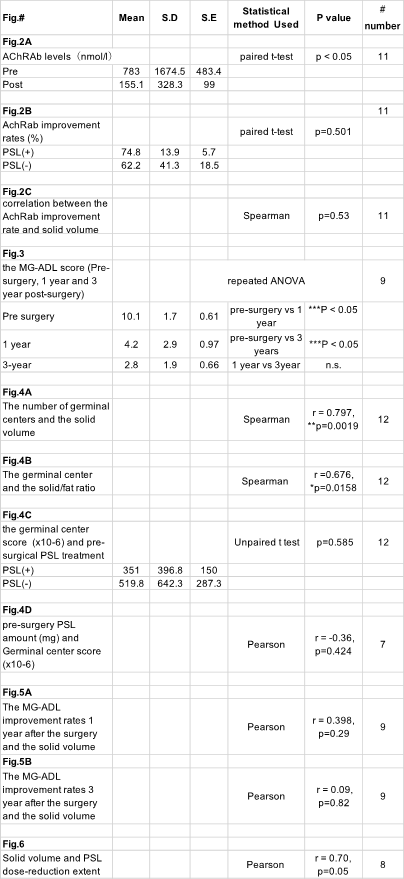

Supplement: S1 Table — (TIF) [file pone.0239756.s001.tif]
