# Supplementary material for: Prediction of improvement after extended thymectomy in non-thymomatous myasthenia gravis patients
Source: PLoS One. 2020 Oct 5;15(10):e0239756. doi: 10.1371/journal.pone.0239756 (PMC7535042; doi:10.1371/journal.pone.0239756)
Supplement: Attachment — Submitted filename: R3 20200829 Reviewers comment .docx [file pone.0239756.s002.docx]

PONE-D-20-12048R1
Prediction of improvement after extended thymectomy in non-thymomatous myasthenia gravis patients

Dear Zsanett Szabo
PLOS ONE

We thank you and the reviewers for your thoughtful suggestions and insights, which have enriched the manuscript and produced a better and more balanced account of the research. We hope that the revised manuscript is now suitable for publication in your journal.

Best regards,

Mitsuteru Yoshida

1. Thank you for including a Supporting Information file containing your data at the end of your manuscript. For composition purposes, kindly provide this as an individual file in Editorial Manager.
   Answer(1)

Thank you for your comments.

We provided the Supporting Information file as the indivisual file in Editorial Managwer and submitted it.

2. Thank you for your response to our recent editorial requests and for providing an updated 'Response to Reviewers' document. At this time, we ask that you please include some of the information that you outlined in the 'Response to Reviewers' document in the Methods section of the manuscript.
Specifically, we ask that you please state that the IRB waived the requirement for informed consent for patients who could not be contacted.
Answer (2)

Thank you for your comments.

We added the information for IRB in the Methods.

“ The study design was approved by the appropriate ethics review board of Tokushima University Hospital (approval number: 3383-1). This study design is a retrospective review of patient medical reviews. The specific source of the medical records is the electronic medical records of Tokushima University Hospital. We could contact some patients; hence, written informed consent could be obtained from these participants. On the other hand, for the patients which we could not contact, we published the information disclosure documents on the home page of Tokushima University Hospital. The IRB specifically waived the requirement for informed consent for patients who could not be contacted. We determined the abovementioned procedures about informed consent according to the Japanese governmental guidelines and have received approval from the Ethics Committee of Tokushima University Hospital. The IRB guideline is equivalent to the guideline of the Ministry of Education, Culture, Sports, Science and Technology and the Ministry of Health, Labor, and Welfare in Japan.”

(Page.8, line 3 – 16)
